# Case report: Treatment of psoriasiform dermatitis in patients with malignancy

**DOI:** 10.3389/fmed.2024.1363405

**Published:** 2024-04-03

**Authors:** Jinzhu Mao, Na Du, Yuanyuan Jia, Qiuyu Mao, Jingyi Yang, Yiwen Zhang, Yueyue Li, Lei Cao, Wei Min

**Affiliations:** ^1^Department of Dermatology, The First Affiliated Hospital of Soochow University, Suzhou, China; ^2^Department of Dermatology, Minhang Hospital, Fudan University, Shanghai, China; ^3^Jiangsu Institute of Clinical Immunology, The First Affiliated Hospital of Soochow University, Suzhou, Jiangsu, China; ^4^Jiangsu Key Laboratory of Gastrointestinal Tumor Immunology, The First Affiliated Hospital of Soochow University, Suzhou, Jiangsu, China; ^5^Jiangsu Key Laboratory of Clinical Immunology, Soochow University, Suzhou, Jiangsu, China

**Keywords:** psoriasiform dermatitis, Janus kinase inhibitor, abrocitinib, PDE-4 inhibitor, apremilast

## Abstract

Psoriasis and atopic dermatitis (AD) are prevalent inflammatory skin disorders, each stemming from diverse factors, and characterized by recurring episodes. In certain complex cases, the clinical and pathological features exhibit overlapping and atypical characteristics, making accurate clinical diagnosis and targeted treatment a challenge. Psoriasiform dermatitis is the term used to describe such cases. Moreover, when patients have a history of malignancy, the situation becomes even more intricate, resulting in limited treatment options. Biologic therapies have transformed the management of immune-mediated inflammatory diseases, including psoriasis and AD. Meanwhile, the safety of biologics in special populations, especially among patients with a history of malignancy, should be underlined. The selective Janus kinase 1 (JAK1) inhibitor abrocitinib has been approved for the treatment of AD and has showed satisfying efficacy and safety in the treatment of psoriasis in clinical trials. Although unreported, JAK1 inhibitors are thought to have the potential to increase the risk of potential tumors. Apremilast, an oral phosphodiesterase (PDE)-4 inhibitor, is approved for moderate to severe plaque psoriasis. It has been investigated for its efficacy in AD, and is not contraindicated in malignancy. This report presents three cases of psoriasiform dermatitis in patients with a history of malignancy, showcasing significant improvement following treatment with systemic glucocorticoid, abrocitinib, or apremilast.

## Case series

### Case 1

**FIGURE 1:**
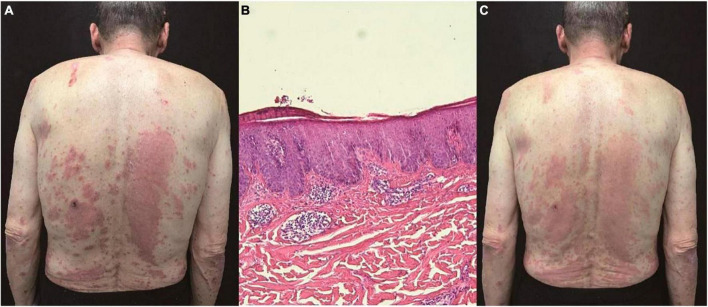
**(A)** Prominent erythema, papules, and plaques distributed across the trunk, limbs, and scalp, all accompanied by adherent white scales. **(B)** Parakeratosis, thickening of the acanthosis cell layer, and a perivascular infiltrate comprising lymphocytes and eosinophils (hematoxylin and eosin, ×100). **(C)** Noticeable improvement of the skin lesions within 2 weeks.

A 72-year-old male patient presented with a 10-year history of scattered erythema accompanied by scales and pronounced pruritus, which progressively extended from the lower extremities to the entire body. Approximately 8 years ago, the patient underwent a skin biopsy with undisclosed results. Over the past few years, intermittent use of topical glucocorticoids and calcipotriol provided temporary relief, but the rash would recur upon discontinuation. Despite recent use of oral antihistamines and topical clobetasol for the past 2 months, pruritus persisted. Consequently, on 16th March 2023, the patient initiated treatment with dupilumab. However, after 4 days, he experienced aggravated erythema and pruritus, prompting him to seek further medical intervention. Notably, the patient had a one-year history of gastric cancer, which had been managed with surgical intervention and chemotherapy. Dermatological examination revealed prominent erythema, papules, and plaques distributed across the trunk, limbs, and scalp, all accompanied by adherent white scales (Figure 1A). The Auspitz sign exhibited a suspicious positive response (±). A subsequent biopsy demonstrated features consistent with psoriasiform dermatitis, including parakeratosis, thickening of the acanthosis cell layer, and a perivascular infiltrate comprising lymphocytes and eosinophils (Figure 1B). Treatment commenced with methylprednisolone at a daily dose of 40 mg, followed by a tapering regimen, resulting in noticeable improvement of the skin lesions within approximately 2 weeks. Subsequently, the patient received oral apremilast, which maintained lesion stability without recurrence after 6 months (Figure 1C).

### Case 2

**FIGURE 2:**
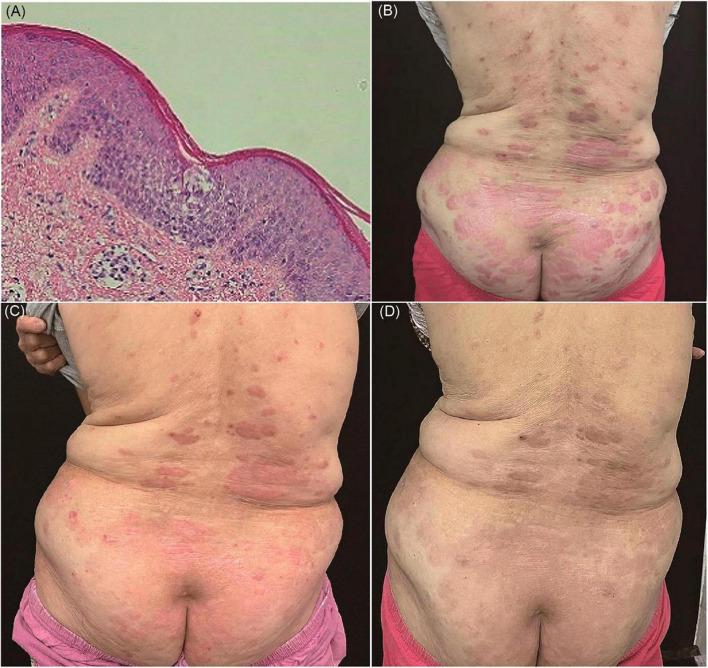
**(A)** Focal sponge edema, lymphocyte and eosinophil infiltration around blood vessels, and without Munro microabscess (hematoxylin and eosin, ×100). **(B)** Erythema and silver scale on the back. **(C)** After treatment of oral JAK1 inhibitor, there is marked remission of skin lesions within 2 weeks. **(D)** The clinical lesions almost disappeared during 12 weeks’ JAK1 inhibitor.

A 69-year-old female patient presented to our dermatology department with a chief complaint of erythema and silver scaling persisting for 6 years on her back, buttocks, and lower limbs, accompanied by severe pruritus. The Auspitz sign elicited a potentially positive response (±). The patient had a history of gastric cancer for 5 years and had undergone surgical treatment. There were no personal or family histories of psoriasis or allergic disease. The diagnosis of psoriasis was established based on the presence of characteristic clinical features. However, the patient exhibited poor response to previous standard treatment modalities, including topical corticosteroids, calcipotriene, and oral acitretin. Unexpectedly, the histopathological examination revealed focal sponge edema, with a limited amount of lymphocyte and eosinophil infiltration surrounding blood vessels in the superficial and mid-dermis, without the presence of Munro microabscesses, which deviated from the typical pathological manifestations of psoriasis (Figure 2A).

Laboratory investigations demonstrated an elevated level of serum IgE (82.61 kU/L; normal range < 60 kU/L) and eosinophils (0.19 × 109/L, 3%). Consequently, dermatitis was considered as a plausible diagnosis. Subsequently, the patient received systemic corticosteroid therapy, resulting in symptom improvement; however, the symptoms resurfaced upon discontinuation (Figure 2B). To explore possible histological changes, a second skin biopsy was performed, which exhibited similar pathological characteristics to the previous biopsy. Considering the patient’s advanced age and mild hepatic and renal dysfunction, conventional immunosuppressants such as methotrexate were deemed unsafe due to potential severe side effects.

As of April 2022, the patient commenced treatment with dupilumab, starting with an initial dose of 600 mg followed by 300 mg every 2 weeks. Surprisingly, this resulted in the exacerbation of skin lesions, characterized by increased erythema and thickened scales within 2 months. Given the patient’s history of malignancy, we sought consultation from the oncologist to assess and verify the inactivity of the tumor. We engaged in communication with the patient and her families and evaluated the risks and benefits associated with JAK inhibitors. Consequently, the patient was transitioned to oral JAK1 inhibitor (abrocitinib) therapy since June 2022, with a daily dosage of 100 mg. Remarkably, the patient experienced significant improvement in itching on the first day of treatment, accompanied by marked remission of skin lesions (Figure 2C). Throughout the 12-week treatment duration and after drug withdrawal, no clinical or biological side effects were observed, and the pathogenic condition remained stable (Figure 2D).

### Case 3

A 68-year-old male patient presented with an 8-month history of scaly plaques affecting his extremities. Three months prior, he received a diagnosis of psoriasis vulgaris and was prescribed topical corticosteroids and apremilast, which effectively alleviated his symptoms. However, 1 month ago, he experienced an exacerbation of skin lesions, accompanied by lower limb swelling and joint pain, following self-discontinuation of apremilast. The patient had a medical history of liver cancer and had been receiving treatment with Toripalimab since May 2021. Although the rash was initially suspected to be a drug reaction, it persisted even after discontinuing the medication.

**FIGURE 3:**
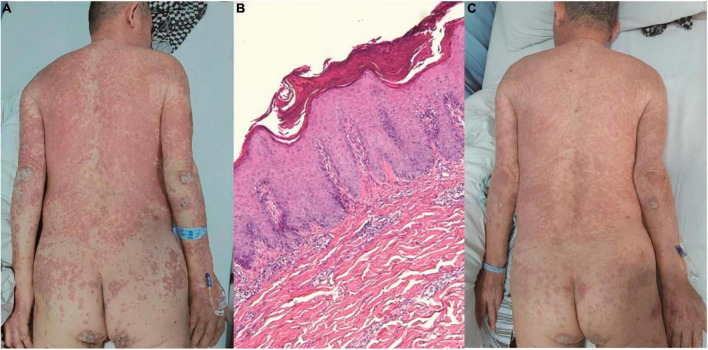
**(A)** Erythema, papules, and plaques covered with white scales on the trunk and extremities. **(B)** Hyperkeratosis with parakeratosis, thickening of the epidermal spinous layer, infiltration of inflammatory cells into the epidermis, dilatation of dermal papillary capillaries, and perivascular infiltration of lymphohistiocytes in the superficial dermis (hematoxylin and eosin, ×100). **(C)** Noticeable improvement of the skin lesions within 2 weeks.

Upon examination, the patient presented with erythema, papules, and plaques covered with white scales on the trunk and extremities. The Auspitz sign elicited a positive response (Figure 3A). Histopathological analysis of a skin biopsy revealed hyperkeratosis with accompanying parakeratosis, loss of the granulosa layer, thickening of the epidermal spinous layer, infiltration of inflammatory cells into the epidermis, dilatation of dermal papillary capillaries, and perivascular infiltration of lymphohistiocytes in the superficial dermis (Figure 3B). Based on the distribution of the skin lesions and the findings from the skin biopsy, the diagnosis of psoriasiform dermatitis was established.

Initially, treatment with apremilast and acitretin was initiated. However, the response to these two medications was relatively slow, and the patient’s joint pain was prominent. Consequently, a low dose of dexamethasone was added. Remarkably, within 2 weeks, the patient’s joint symptoms and skin lesions were effectively controlled, leading to his discharge from the hospital (Figure 3C).

## Discussion

Psoriasis and AD are inflammatory skin diseases with distinct clinicopathologic presentations driven by different immunologic mechanisms. While Th17 and Th22 cells are typically associated with psoriasis, AD is characterized by abnormal activation of Th2 cells (1). However, emerging evidence suggests that these seemingly distinct inflammatory pathways can intersect and contribute to the coexistence and conflicting relationships observed in both psoriasis and AD. Consequently, distinguishing between psoriasis and AD, especially when clinical and histological findings overlap, can be challenging (2). In such cases, the term “psoriasiform dermatitis” is employed to describe variable eczema-like histopathological changes with a psoriasiform appearance (3–5). Histologic features overlapping with psoriasis include edema of the papillary dermis and the presence of Munro microabscesses, while AD-associated features encompass neutrophil and plasma exudation in parakeratosis, lymphocytic exocytosis in the epidermis, full-thickness spongiosis, or spongiotic vesicle formation. Despite the successful use of biologics targeting pathogenic molecules in psoriasis and AD, treatment resistance has been observed in patients presenting with psoriasiform dermatitis (6).

In this report, we presented three cases with complex clinical and pathological manifestations. Table 1 summarized the three cases. All patients exhibited erythema, silver scale, and a positive Auspitz sign, suggestive of psoriasis. However, the histopathological findings supported a diagnosis of psoriasiform dermatitis. In cases 1 and 2, treatment with dupilumab proved ineffective and, in some instances, worsened the condition. Conversely, systemic steroids demonstrated significant efficacy in all patients, underscoring their potential role in controlling psoriasiform dermatitis, despite not being the standard treatment for psoriasis vulgaris. In case 2, the subsequent use of oral JAK1 inhibitor led to rapid improvement of clinical symptoms. Notably, no adverse reactions or fluctuations in the pathogenic condition were observed during the 12-week treatment or after drug withdrawal. In cases 1 and 3, apremilast was effective in achieving remission and disease stabilization. These findings demonstrate the efficacy of abrocitinib and apremilast in treating psoriasiform dermatitis.

**TABLE 1:** Clinical characteristics of the patients.

Patient	Sex/age	Duration	History of malignancy	Histological examination	Previous therapies	Management	Outcome
1	M/72	10 years	Gastric cancer	Parakeratosis, thickening of the acanthosis cell layer, and a perivascular infiltrate comprising lymphocytes and eosinophils	Dupilumab	Cs, apremilast	Improvement
2	F/69	6 years	Gastric cancer	Focal sponge edema, lymphocyte and eosinophil infiltration around blood vessels, and without Munro microabscess	Dupilumab	Abrocitinib	Improvement
3	M/68	8 months	Liver cancer	Hyperkeratosis with parakeratosis, thickening of the epidermal spinous layer, infiltration of inflammatory cells into the epidermis, dilatation of dermal papillary capillaries, and perivascular infiltration of lymphohistiocytes in the superficial dermis	Apremilast, acitretin	Apremilast, acitretin, added with Cs	Improvement

M, male; F, female; Cs, corticosteroids.

Dupilumab, an interleukin (IL)-4 and IL-13 receptor monoclonal antibody, is approved for the treatment of asthma, atopic dermatitis, and chronic rhinosinusitis. Dupilumab showed no difference in efficacy or safety when treating AD patients with a history of malignancy (7). However, reports have indicated the emergence of psoriasiform dermatitis following dupilumab treatment for AD (5, 6). It is hypothesized that dupilumab-associated Th2 immunomodulation inadvertently activates Th1 and Th17 pathways implicated in psoriasis pathogenesis. In our cases, dupilumab proved ineffective in treating psoriasiform dermatitis and even exacerbated the condition, suggesting distinct pathogenic mechanisms between psoriasiform dermatitis and AD.

Abrocitinib, a JAK1 selective inhibitor, acts on the JAK/signal transducers and activators of transcription pathway, which mediates the effects of various cytokines such as IL-4, IL-5, IL-13, IL-31 and IL-22. These cytokines play roles in keratinocytes, immune cells, and peripheral sensory neurons, contributing to inflammation and itching in inflammatory skin diseases, including dermatitis (8, 9). JAK1 inhibitors may also impair IL-23 receptor function, inhibiting the infiltration of skin immune cells and the production of IL-17 and IL-22. The IL-23/Th17 pathway is crucial in psoriasis pathogenesis, indicating the therapeutic potential of JAK inhibitors (10). Due to their extensive anti-inflammatory actions, JAK inhibitors have demonstrated efficacy in the treatment of various inflammatory skin diseases, including AD and psoriasis (11, 12). In our cases, abrocitinib proved effective in treating psoriasiform dermatitis. While JAK inhibitors can block interferons and natural killer cells important to tumor surveillance, multiple large studies of patients with inflammatory diseases have failed to demonstrate an increased risk of cancer (13). For the few cases of malignancies reported in clinical trials, causal relationships cannot be established (14). Nevertheless, it is necessary to cooperate with an oncologist to assess the risks and benefits of using JAK inhibitors. Adequate communication with patients and families is also important. Moreover, JAK inhibitors are not suggested to be used in patients with a history of malignancy < 5 years.

Apremilast, an oral PDE-4 inhibitor, is FDA-approved for psoriatic arthritis and moderate to severe plaque psoriasis. PDE-4 is widely expressed in immune cells such as macrophages, lymphocytes, and natural killer cells, as well as non-hematopoietic cells like keratinocytes and synovial fibroblasts. In peripheral blood mononuclear cells, PDE-4 inhibition reduces the production of pro-inflammatory cytokines, including tumor necrosis factor α, IL-12/23, IL-12, and IL-2, while upregulating the anti-inflammatory cytokine IL-10. Due to its broad cellular targets and relative tolerability, apremilast has been investigated for its efficacy in chronic inflammatory diseases, including AD and different psoriasis variants (15). In a recent report documenting the real-life utilization of apremilast in patients diagnosed with both psoriasis and cancer, apremilast demonstrated an improvement in quality of life, while maintaining a favorable safety profile (16, 17). Our cases demonstrated the effectiveness of apremilast in achieving remission and maintaining stability without recurrence. Apremilast is generally considered safe, with few serious adverse effects reported, consistent with our cases.

In our cases, all three patients shared a history of digestive system malignancy. Paraneoplastic dermatitis, which occurs in various malignancies, presents with diverse clinical features. Bazex syndrome, a rare cutaneous paraneoplastic disorder, is characterized by psoriasiform lesions on acral areas (18). Notably, these psoriasiform eruptions often precede the cancer diagnosis by several months. Symptomatic improvement typically follows the treatment of the underlying malignancy. However, in our cases, the skin manifestations appeared after the discovery of the tumors, distinguishing them from Bazex syndrome. Further investigations are needed to explore potential molecular and cellular links between the described skin features and tumors.

In recent years, our understanding of the pathogenesis of inflammatory skin disorders has significantly advanced, leading to revolutionary targeted therapies in dermatology, such as JAK inhibitors and PDE-4 inhibitors. However, the presence of concomitant comorbidities often represents a limit to the treatment (19). The findings indicate that a history of malignancies does not constitute an absolute contraindication for biologics. The treatment of patients with malignancy emphasized a nuanced assessment of benefits and risks (20). Further long-term observations are necessary to ensure the safety and stability following the withdrawal of abrocitinib and apremilast. Given the multifactorial nature of pathogenesis in skin disorders, we anticipate more studies exploring alternative targets for refractory inflammatory skin diseases with multifunctional potential.

## Patient consent

Consent for the publication of all patient photographs and medical information was provided by the authors at the time of article submission to the journal stating that all patients gave consent for their photographs and medical information to be published in print and online and with the understanding that this information may be publicly available.

## Data availability statement

The original contributions presented in this study are included in this article/supplementary material, further inquiries can be directed to the corresponding authors.

## Ethics statement

Ethical approval was not required for the studies on humans in accordance with the local legislation and institutional requirements because only commercially available established cell lines were used. Written informed consent was obtained from the individual(s) for the publication of any potentially identifiable images or data included in this article.

## Author contributions

JM: Writing – original draft, Writing – review & editing. ND: Writing – original draft, Writing – review & editing. YJ: Data curation, Writing – original draft. QM: Formal analysis, Writing – review & editing. JY: Investigation, Writing – original draft. YZ: Methodology, Writing – review & editing. YL: Project administration, Writing – review & editing. LC: Supervision, Writing – original draft, Writing – review & editing. WM: Supervision, Writing – original draft, Writing – review & editing.
